# The intervention effect of physical and mental exercise on young adults internet addiction disorder: a systematic review and Bayesian model analysis

**DOI:** 10.3389/fpubh.2025.1670168

**Published:** 2025-10-02

**Authors:** Shiguan Jia, Hao Wang, Dengshan Chu, Jiayi Yao, Haozhe Wang, Wenjia Chen, Dazhong Zhang, Wenzhong Zhang

**Affiliations:** ^1^School of Physical Education, China University of Mining and Technology, Xuzhou, Jiangsu, China; ^2^School of Physical Education and Coaching Science, Capital University of Physical Education and Sports, Beijing, China; ^3^Department of Physical Education, Tianjin Medical University, Tianjin, China

**Keywords:** internet addiction disorder, physical and mental exercise, dose-response relationship, young adults, exercise intervention

## Abstract

**Background:**

As a behavioral addiction, internet addiction disorder has become a global problem that seriously affects people’s mental health. Although physical and mental exercise is believed to help alleviate related symptoms, there is currently a lack of systematic research evaluating the intervention effects of different physical and mental exercises on internet addiction disorder.

**Objective:**

To systematically evaluate the effects of different physical and mental exercise modes and amounts on the alleviation of symptoms of internet addiction.

**Method:**

Randomized controlled trials (RCTs) on the effects of different physical and mental exercise modes on internet addiction published between October 2000 and February 2025 were retrieved through PubMed, Web of Science, EBSCO, Cochrane Library, and CNKI systems. After independently screening literature, extracting data, and evaluating the risk of bias in the included studies by two independent researchers, a random effects model was used for meta-analysis using RevMan 5.4 and Stata 19.0 software. Perform dose-response analysis using R software.

**Results:**

Twenty-four randomized controlled trials involving 1,711 participants were included. Compared with the control group, all physical and mental exercise methods significantly improved symptoms of internet addiction disorder (SMD = −1.63, 95% CI: −2.04, −1.22). Mindfulness meditation showed the strongest effect (SMD = −2.04, 95% CI: −3.23, −0.85). The study determined a non-linear U-shaped dose-response relationship, with the best effect occurring at 730 MET min/week.

**Conclusion:**

This study provides theoretical support for non pharmacological interventions to improve symptoms of internet addiction disorder. For those who hope to improve their internet addiction through physical and mental exercise, mindfulness meditation is the first choice. In addition, controlling the exercise dose within the optimal range (e.g., 730 MET min/week) can significantly enhance the intervention effect.

**Systematic review registration:**

https://www.crd.york.ac.uk/PROSPERO/view/CRD42025631096, CRD42025631096

## Background

Internet Addiction Disorder (IAD) is a kind of behavioral addiction, which refers to a mental and behavioral disorder caused by uncontrolled Internet use. It usually shows clinical characteristics such as overuse, withdrawal symptoms, tolerance and negative effects (1). In 2013, internet gaming disorder, a subtype of IAD, was officially included for the first time by the American Psychiatric Association (APA) in the 5th edition of the Diagnostic and Statistical Manual of Mental Disorders, Part 3, “List of Subjects for Further Research” (2). IAD affects approximately 14.22% of the global population, with significant regional variations (0.8–26.7%) (3). College students face particularly high risk due to two key factors: (1) developmental vulnerability—newfound internet freedom without fully matured self-regulation abilities, and (2) environmental pressures related to academics and social identity formation (4, 5). Research demonstrates that IAD severity positively correlates with the magnitude of adverse physical and mental health outcomes (6). As an emerging mental health issue, IAD has not yet formed a mature and complete treatment system. Most current treatments are adapted from approaches used for substance addictions, such as alcohol and drug dependencies (7).

Mind–body exercise represents a promising intervention approach for IAD, as these practices enhance self-regulation, stress management, and impulse control—all key factors in addiction recovery. Király et al. emphasized in the consensus guidelines on internet use during the COVID-19 pandemic that structured physical activity and psychosomatic interventions can help prevent and alleviate IAD symptoms (8). For prevention, Zhu et al. found that regular physical activity significantly reduces problematic gaming behavior, particularly among young adults (9). Similarly, Throuvala et al.’s systematic review indicates that school programs incorporating mindfulness and physical activity effectively prevent IAD in young adults, with high compliance and sustainability (10). For treatment applications, mind–body exercise shows promise for IAD intervention. However, two key limitations exist in current research: first, the lack of systematic comparison between different mind–body exercise methods prevents determination of optimal clinical interventions (11); second, insufficient quantitative research on dose-response relationships makes it difficult to establish optimal exercise prescriptions (including frequency, intensity, and duration) (12). These gaps limit evidence-based application and personalized prescription development of mind–body exercise as a non-pharmacological intervention for IAD.

In summary, to our knowledge, this study is among the first to systematically evaluate the differences in the effectiveness of seven mind–body exercise methods: Tai Chi, Baduanjin, DanceSports, Mindfulness, Aerobicdance, Yoga, and Qigong, in improving IAD symptoms. Through dose-response analysis, we quantify the optimal intervention parameters for IAD symptom improvement. This provides empirical basis for large-scale promotion of IAD intervention strategies and advances personalized, precise IAD treatment approaches.

## Materials and methods

### Registration

This investigation adhered to the Systematic Review and Meta-Analysis PRISMA reporting framework and received advance registration in the PROSPERO database under identification code CRD42025631096. This pre registration step ensures transparency of the method and compliance with standardized review practices.

### Search strategy

Search protocols for identifying IAD and physical-mental exercise interventions were formulated utilizing Medical Subject Headings (MeSH) terminology combined with Boolean logic operators. The comprehensive retrieval methodology incorporated both standardized MeSH classifications and relevant keywords. Box 1 presents the detailed query structure implemented within the PubMed database platform.

BOX 1 **PubMed search strategy.**

#1 (“Mind”[Title/Abstract] OR “body exercise”[Title/Abstract] OR “Mind–body exercise”[Title/Abstract] OR “Taichi”[Title/Abstract] OR “Baduanjin”[Title/Abstract] OR “Mindfulness”[Title/Abstract] OR “Dance Sport”[Title/Abstract]) OR “Qigong”[Title/Abstract] OR “Yoga”[Title/Abstract] OR “Aerobicdance”[Title/Abstract] AND “Internet Addiction Disorder”[MeSH Terms]

#2 “Sports”[Title/Abstract] OR “Sport”[Title/Abstract] OR “Athletics”[Title/Abstract] OR “Athletic”[Title/Abstract]

#3 #1 OR #2

#4 “Internet Addiction Disorder”[Title/Abstract] OR “Addiction Disorder, Internet”[Title/Abstract] OR “Addiction Disorders, Internet”[Title/Abstract] OR “Disorder, Internet Addiction”[Title/Abstract] OR “Disorders, Internet Addiction”[Title/Abstract] OR “Internet Addiction Disorders”[Title/Abstract] OR “Internet Addiction”[Title/Abstract] OR “Addiction, Internet”[Title/Abstract] OR “Addictions, Internet”[Title/Abstract] OR “Internet Addictions”[Title/Abstract] OR “Internet Gaming Disorder”[Title/Abstract] OR “Disorder, Internet Gaming”[Title/Abstract] OR “Disorders, Internet Gaming”[Title/Abstract] OR “Gaming Disorder, Internet”[Title/Abstract] OR “Gaming Disorders, Internet”[Title/Abstract] OR “Internet Gaming Disorders[Title/Abstract] OR Smartphone Addiction[Title/Abstract] OR Addiction, Smartphone[Title/Abstract] OR Addictions, Smartphone[Title/Abstract] OR Smartphone Addictions[Title/Abstract] OR Social Media Addiction[Title/Abstract] OR Addiction, Social Media[Title/Abstract] OR Addictions, Social Media[Title/Abstract] OR Media Addiction, Social[Title/Abstract] OR Media Addictions, Social[Title/Abstract] OR Social Media Addictions[Title/Abstract]

#5 “randomized controlled trial”[Publication Type] OR “controlled clinical trial”[Publication Type]

#6 #3 AND #4 AND #5

### Inclusion and exclusion criteria

#### Literature inclusion criteria

This investigation adhered rigorously to the PRISMA guidelines (13), establishing comprehensive search protocols and selection criteria based on PICOS methodology in evidence-based practice. Only randomized controlled trials examining how structured exercise interventions impact Internet Addiction Disorder among young adults (18–29 years) were selected for analysis. Experimental cohorts participated in various movement-based or contemplative practices—including Tai Chi, Baduanjin, Mindfulness, Dancesports, Aerobic dance, Yoga, or Qigong—while control conditions included no treatment, treatment as usual, or waitlist controls without structured mind–body interventions. Assessment utilized identical IAD measurement instruments across both groups, administered pre- and post-intervention. Intervention efficacy was determined when the differential calculation between baseline and concluding measurements yielded negative values, Indicating an improvement in symptoms of internet addiction.

#### Exclusion criteria

Non RCT experiments; animal studies; descriptive research, reviews, secondary analyses, conference abstracts, and duplicated publications; studies lacking standard statistical presentation (x ± s) or with unextractable metrics; research with confounding interventions making isolation of treatment effects impossible; and studies with incomplete data reporting or unconvertible measurements (Table 1).

**Table 1:** PICOS-based eligibility criteria (participation, intervention comparison, outcomes, and study design).

PICOS	Criteria
Participants	Young Adults
Intervention	Mind–Body Exercise
Comparison	Regular physical activity group
Outcome	Internet Addiction Disorder
Study design	Randomized controlled trial

### Literature screening and data extraction

After excluding duplicate literature using NoetExpress V4. X software, two researchers independently screened based on inclusion and exclusion criteria and cross checked the results. Disagreements were resolved through discussion until consensus was reached, with a third researcher serving as arbitrator when necessary. Cohen’s kappa coefficient was calculated to assess inter-rater reliability during the screening process, achieving *K* = 0.94. The data extraction content mainly includes: (1) publication details (title, first author); (2) Participant characteristics (sample size, age, gender, etc.); (3) Intervention standards (type, duration, frequency, pre- and post test scores of the intervention group and control group on the IAD Scale, etc.); (4) The inter rater reliability of the measurement tools and results, as well as the data encoding, is 94%. Any differences are resolved through re examination and consensus meetings. Use the Cochrane Bias Risk Tool for randomized controlled trials to assess the risk of bias.

After extracting the mean (M) and standard deviation (SD) of the scale measurement results before and after intervention experiments from various literature, two researchers calculated the difference in mean values between pre- and post-intervention measurements. For studies not reporting the pre-post correlation coefficient, we assumed *R* = 0.5 following recommendations from the Cochrane Handbook (14). This assumption was tested in sensitivity analyses using alternative values (*R* = 0.4, 0.6) to assess the robustness of our findings. When studies reported standard error (SE) rather than standard deviation, we converted values using standard formulae. we converted it to *R* = 0.5, as shown below:


$$R=\left(SD^{2}baseline+SD^{2}final-SD^{2}change\right)/2\times SDbaseline\times SDfinal$$

This study referred to meta-analyses by other scholars, and the results were similar, with *R*-values of 0.5 (15) and. After sensitivity analysis, the study ultimately chose *R* = 0.5. In addition, if the original research data is provided as SE, SD is obtained by multiplying the SE of the mean by the square root of the sample size, where *N* is the sample size:


$$SD=SE\times \sqrt{N}$$

### Data encoding and management

According to pre-defined categories, the included exercise intervention programs are coded as TC (Tai Chi), BDJ (Baduanjin), MF (Mindfulness), DS (DanceSports), QG (Qigong), AD (Aerobicdance), YG (Yoga). We quantified intervention intensity using metabolic equivalent of task (MET) values, where 1 MET represents energy expenditure at rest. MET values were assigned to each intervention based on the 2024 Adult Physical Activity Guidelines (16), the American College of Sports Medicine Exercise Testing and Prescription Guidelines (17), and clinical exercise dosage literature (18). Specifically, we assigned: TC = 3.0 METs, BDJ = 2.5 METs, MF = 1.5 METs, DS = 4.5 METs, QG = 2.5 METs, AD = 5.0 METs, and YG = 3.0 METs. For mindfulness, which is not traditionally measured in METs, we used values from similar seated, focused attention activities. Daily dose was calculated as MET × daily intervention time, and weekly dose as weekly frequency × daily dose. To ensure network connectivity for analysis, we classified interventions into standardized weekly dosage categories: 0 (CG), 100, 200, 300, 400, 500, 600, 700, 1,000, 1,200 METs·min/week.

### Data analysis

#### Meta-analysis

Meta analysis was conducted on the data extracted from the literature using Stata19.0 software. Due to the different measurement tools used for the same indicator in the included literature and the fact that the experimental data were continuous variables, standardized mean difference (SMD) and 95% confidence interval (95% CD) were used as effect measures to merge the effect sizes (19). When *p* < 0.05, there is a significant difference between the experimental group and the control group, indicating that the results of the meta-analysis are statistically significant (20). When testing heterogeneity, if the heterogeneity *I*^2^ is less than 50%, the fixed effects model is used for analysis. If *I*^2^ is greater than 50%, the random effects model is used for analysis (21). Use subgroup analysis, sensitivity analysis, and meta regression analysis to determine whether the included studies are biased or require heterogeneity source testing (22).

#### Dose-response analysis

A Bayesian random-effects framework was employed to evaluate dose-response associations between mind–body exercise interventions and IAD utilizing the “brms” package (version 4.3.1) in R (23, 24). This methodological approach facilitates modeling of nested effect size structures within studies, thereby accounting for statistical dependencies when multiple effect sizes derive from identical participant cohorts. Standardized mean difference (SMD) changes in IAD were modeled through both linear and non-linear specifications (natural spline with 3 or 4 knots) to appropriately adjust for weekly physical activity dosage gradients. Weakly informative prior distributions were specified for the key parameters (overall effect size *μ* [0, 1] and between-study heterogeneity Tau [0, 1]) (25), with sensitivity analyses incorporating alternative prior specifications (μ [0, 0.5] and Tau [0, 0.5]) conducted to examine the robustness of posterior probability distributions. Optimal model selection proceeded via quantitative comparison of expected log pointwise predictive density, effective parameter count, and cross-validation information criteria. In addition, we adopted a refinement strategy of saving results every 40 iterations to optimize the data monitoring process and reduce storage requirements (26). Model convergence assessment utilized the potential scale reduction factor criterion (PSRF < 1.05) complemented by visual examination of Markov Chain Monte Carlo (MCMC) trace plots and posterior density distributions (27). Comprehensive technical specifications regarding MCMC implementation parameters (iteration quantity, burn-in period duration, thinning intervals) are delineated in Supplementary material S1.

#### Inclusion in literature bias risk assessment

For quality assessment of included randomized controlled trials, we applied the Second Edition of Cochrane Risk Bias Tool (RoB 2) through RevMan 5.4 software platform. The assessment procedure involved dual independent evaluations conducted by separate investigators. Their comprehensive analysis examined multiple bias domains: generation of randomization sequences, concealment methods for allocation, blinding protocols for both participants and researchers, measures ensuring outcome assessor blinding, approaches to handling missing outcome information, selective outcome reporting issues, plus additional potential bias sources.

The research quality rating adopts a three-level standard: those who fully meet the standard are marked as “+,” indicating low-risk bias; Those who completely do not meet the criteria are marked as “-,” indicating high-risk bias; Those who do not explicitly describe relevant information in the literature are marked with “?,” indicating unknown risk bias. If the results of two researchers are inconsistent during the evaluation process, the third researcher will participate in the discussion.

## Results

### Data selection

**Figure 1:**
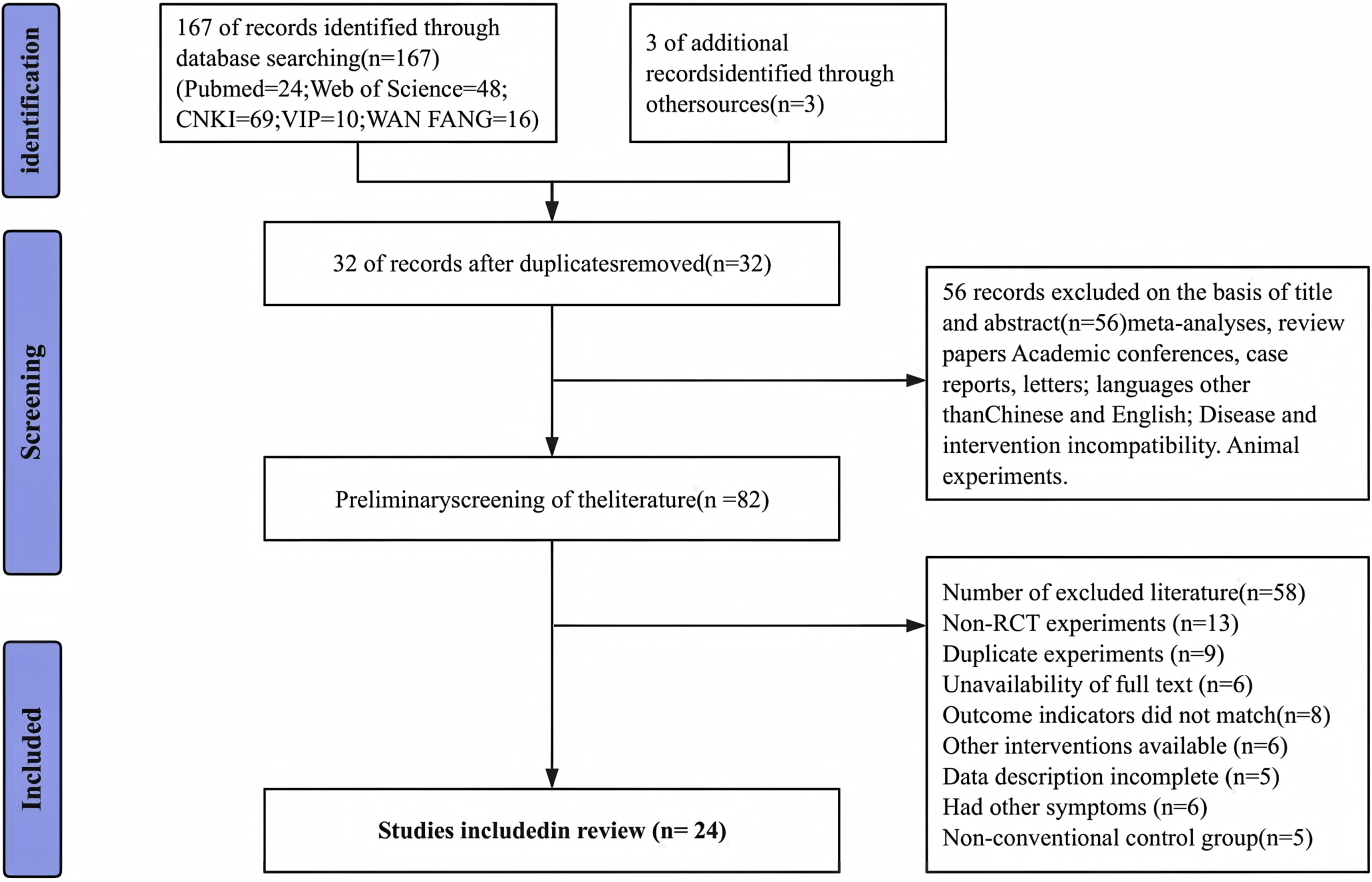
PRISMA research selection process flow chart.

The PRISMA flowchart illustrates the research selection process, as shown in Figure 1. Systematically retrieve databases using pre-defined retrieval strategies. A preliminary search has identified 170 potential studies. After using EndNote software for database management and deleting 32 duplicate studies, there are still 82 studies. After independent screening of titles, abstracts, and complete manuscripts by three researchers, a total of 24 studies were included in the final analysis, including both English and Chinese literature.

### Characteristics included in the study

The study included 24 studies conducted between 2000 and 2025, involving 1,711 young Adults aged 18–24 with a median age of 24. Sports interventions include intervention forms such as Tai Chi, Baduanjin, Mindfulness, Aerobicdance, sports dance, and yoga. Specifically, 143 participants from 5 research groups received Tai Chi, 367 participants from 4 research groups received Baduanjin, 223 participants from 9 research groups received mindfulness meditation, 74 participants from 3 research groups received aerobic fitness exercises, 4 participants from 1 research group received sports dance, 15 participants from 1 research group received yoga, and 31 participants from 1 research group received qigong. The intervention duration ranges from 4 weeks to 16 weeks, with a median duration of 10 weeks. The intervention frequency is 1–10 times per week, with a median frequency of 5.5 times. The duration of each session is 30–150 min, with a median duration of 90 min. The detailed features included in the study are shown in Table 2.

**Table 2:** Characteristics included in the study.

Author	Type of intervention	Sample size (M/F)	T/C	Source	Age (T/C)	Periodicity, frequency and duration of interventions	Scale used for outcome measurement
Yang et al. (28)	Tai chi	25/27	26/26	China	19.6 ± 1.2	16 weeks, 4times/week, 60 min/session	CIAS
Zhang et al. (29)	Tai chi	24/38	31/31	China	20.1 ± 0.75	8 weeks, 3times/week, 60 min/session	IAT
Xiao et al. (30)	Baduanjin	48/17	31/34	China	19.71 ± 1.71	12 weeks, 3times/week, 90 min/session	MPAI
Zhang et al. (31)	Tai chi	24/36	30/30	China	20.1 ± 0.76	8 weeks, 3times/week, 60 min/session	SAS – SV
Lan et al. (32)	Mindfulness	22/32	27/27	China	21.3 ± 1.3	8 weeks, 1/week, 60 min/session	MPIAS
Ren et al. (33)	Dance Sport	6/2	4/4	China	22 ± 2	12 weeks, 3times/week, 90–120 min/session	SCL – 90
Xie et al. (34)	Baduanjin	14/530	274/270	China	–	8 weeks, 10times/week, 20–30 min/times	MPAI
Zhang et al. (35)	Tai chi	4/48	26/26	China	20.5 ± 1.5	8 weeks, 3times/week, 60 min/time	MPAI
Liu et al. (36)	Tai chi	31/34	31/34	China	–	10 weeks, 2times/week, 60 min/session	MPAI
Li et al. (37)	Mindfulness	21/38	28/31	China	20.14 ± 1.32	8 weeks, 1/week, 150 min	MPATS
Lu et al. (38)	Qigong	48/17	31/34	China	19.21 ± 1.02	12 weeks, 2/week, 90 min/session	MPATS
Yang et al. (39)	Aerobicdance	41/31	36/36	China	17.45 ± 2.02	12 weeks, 3times/week, 30 min/session	MPAI
Dai et al. (40)	Mindfulness	–	27/20	China	–	4 weeks, 1times/week, 150 min/session	SAS–C
Liu et al. (41)	Baduanjin	48/17	31/34	China	18.95 ± 0.89	12 weeks, 3/week, 90 min/session	MPAI
Zhang et al. (42)	Mindfulness	13/11	12/12	China	–	6 weeks, 1times/week, 90 min/session	MPAI
Yang et al. (43)	Mindfulness	–	30/30	China	–	4 weeks, 7/week, 20 min/session	SAS–CA
Yu et al. (44)	Aerobicdance	0/60	30/30	China	18.83 ± 0.87	16 weeks, 3/week, 60 min/session	MPAI
Liao et al. (45)	Aerobicdance	–	8/8	China	20.12 ± 1.54	6 weeks, 3times/week, 90 min/session	MPAI
Zhu et al. (46)	Baduanjin	26/34	30/30	China	–	8 weeks, 3times/week, 60 min/session	MPATS
Tadpatrikar and Kumar (47)	Yoga	22/7	15/14	India	22.9 ± 6.5	8 weeks, 7times/week, 35 min/session	S–IAT
Wu et al. (48)	Mindfulness	43/25	34/34	China	20.36 ± 2.14	8 weeks, 5times/week, 60 min/session	PVGUA
Wang et al. (49)	Mindfulness	7/30	22/15	China	20.05 ± 1.05	8 weeks, 7times/week, 90 min/session	MPAI
Ren et al. (50)	Mindfulness	7/12	9/10	China	–	8 weeks, 1times/week, 90 min/session	MPAI
Shen et al. (51)	Mindfulness	27/41	34/34	China	18–22	8 weeks, 1/week, 90 min/session	IAT

T, Treatment group; C, Control group; CIAS, Chinese Internet Addiction Scale (Chen); IAT, Internet Addiction Test (Young); MPAI, Mobile Phone Addiction Index (Leung); SAS-SV, Smartphone Addiction Scale-Short Version; SCL-90, Symptom Checklist-90 (Young’s Internet Addiction Related Psychological Scale); MPATS, Mobile Phone Addiction Tendency Scale for College Students (Xiong); SAS-C, The Smartphone Addiction Scale for College Students (Su Shuang); SAS-CA, Adult Smartphone Addiction Scale (Chen Huan); PVGUA, Pathological Online Game Usage Questionnaire.

### Risk of bias and evidence assessment

#### Risk of bias

**Figure 2:**
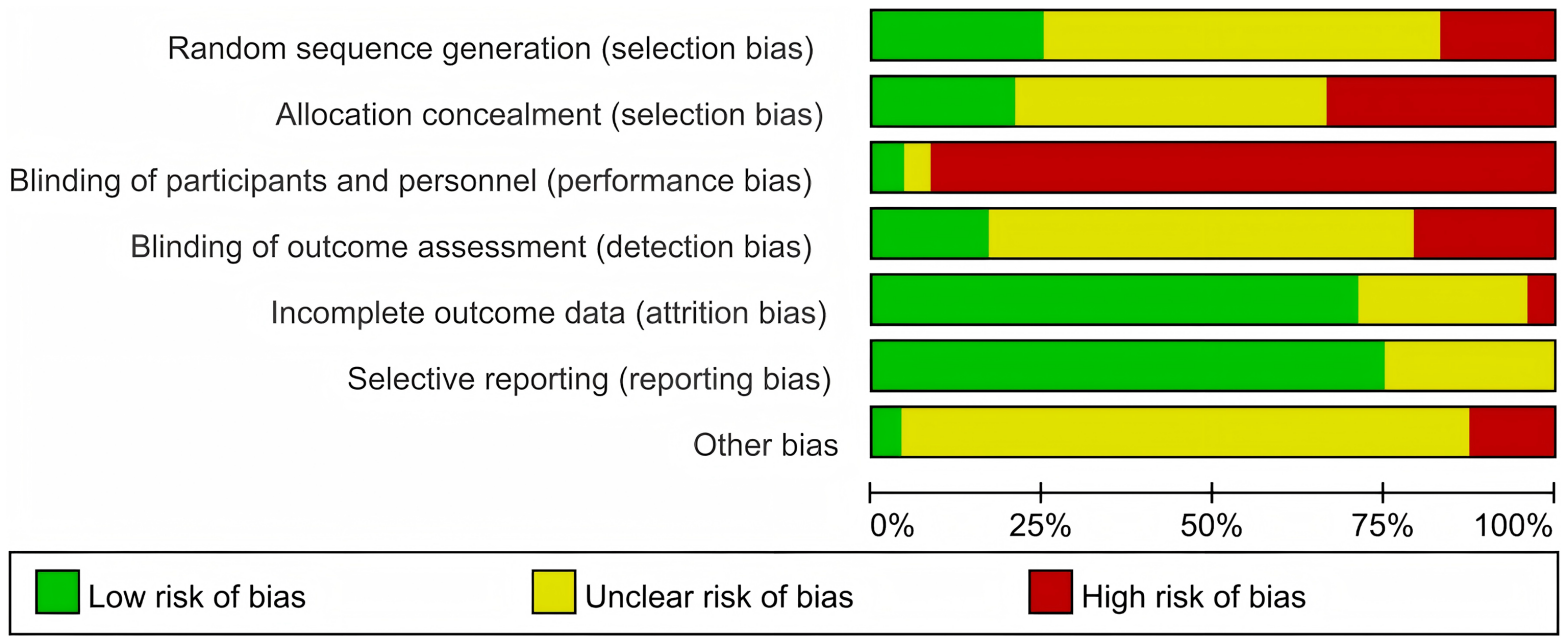
Bar chart of bias risk assessment results for included literature.

**Figure 3:**
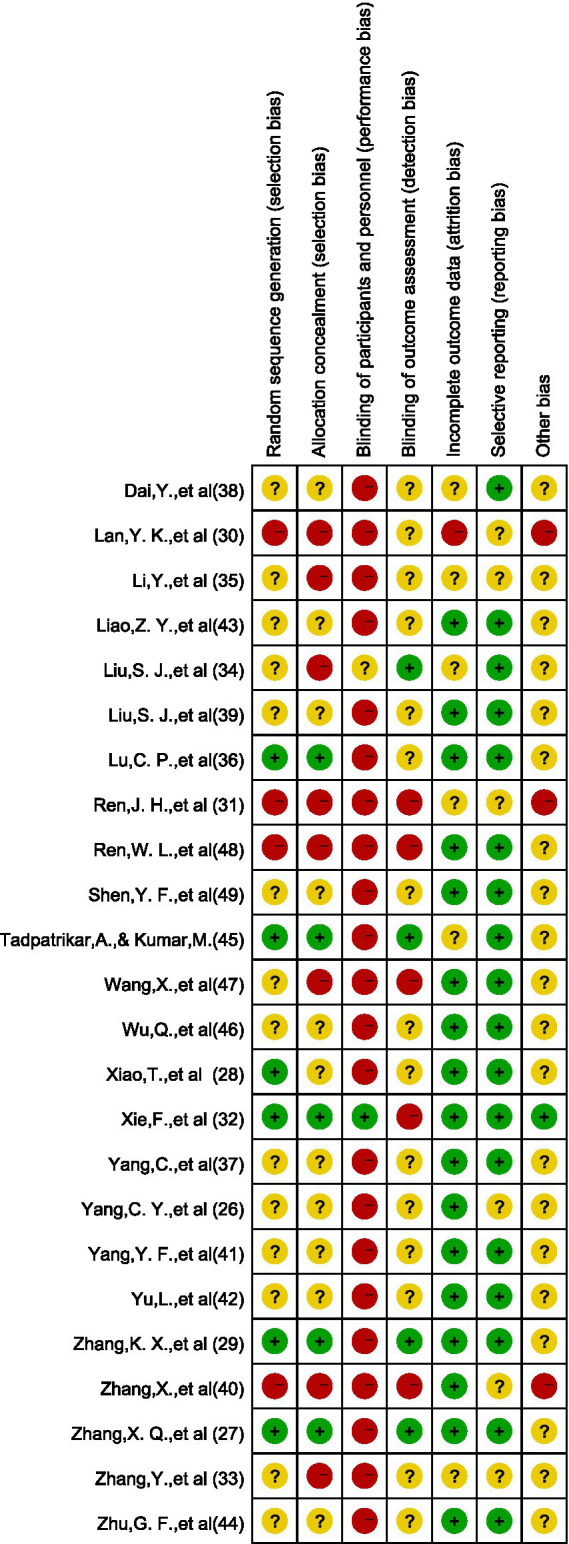
Quality assessment heatmap of bias risk assessment results for included literature.

Figures 2, 3 show detailed information on bias risk assessment. Among the 24 studies included, 3 (12.5%) were assessed as low-risk, 11 (45.8%) had certain issues, and 10 (41.7%) showed high-risk bias. From the perspective of bias types, implementation bias is the most prominent, with about 95% of studies showing a high risk of participant and researcher blinding. This is due to the inherent characteristics of mental health intervention research, as participants and implementers often cannot completely blind intervention types. It is worth noting that the risk of incomplete outcome data (loss bias) and selective reporting (report bias) is relatively low, with approximately 70–80% of studies rated as low-risk, indicating good methodological quality in terms of data integrity and reporting transparency. There is a certain proportion of unclear risk (about 50%) in the blind methods of random sequence generation, allocation concealment, and result evaluation, mainly due to insufficient details in the original research report rather than substantial flaws in the research design.

Overall, although there is heterogeneity in the methodological quality of the included studies, it does not affect the overall reliability and clinical significance of the results of this study.

#### Evidence evaluation

According to the CINeMA system, the overall quality of evidence is assessed as low due to the risk of bias and inconsistency.

Meta analysis of the effect of physical and mental exercise on improving symptoms of young Adults IAD.

**Figure 4:**
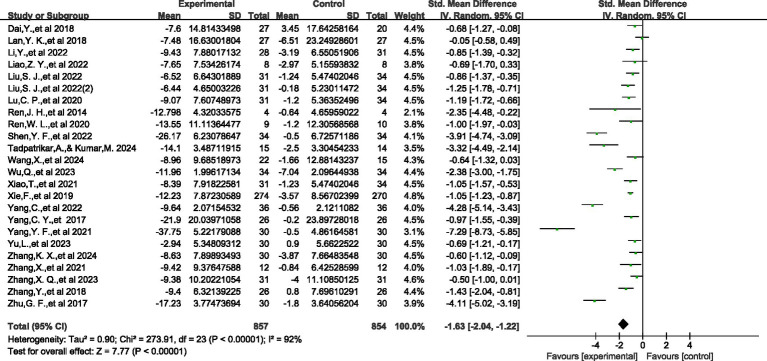
The effect of physical and mental exercise on improving symptoms of IAD.

A meta-analysis of 24 controlled studies was also conducted (*N* = 1,711). The overall results of the meta-analysis are presented in Figure 4. Compared with the control group, we found that the experimental intervention had a significant effect on reducing the primary outcome measure (SMD = −1.63, 95% CI: −2.04, −1.22). The *I*^2^ statistic showed substantial heterogeneity (*I*^2^ = 92%, df = 23, *p* < 0.00001). Analysis of the forest plot shows that all included studies demonstrated negative effect values, indicating a consistent direction of effect for the intervention. The most significant effect was observed in Yang, Y. F., et al., 2021 (SMD = −7.29, 95% CI: −8.73, −5.85), while the smallest effect was found in Lan, Y. K., et al., 2018 (SMD = −0.05, 95% CI: −0.58, 0.49). Given the high heterogeneity, we employed a random-effects model for analysis, with an overall effect test of *Z* = 7.77 (*p* < 0.00001), indicating that the intervention effect was statistically significant.

### Meta analysis on the improvement effect of different physical and mental exercises on Young Adults IAD

**Figure 5:**
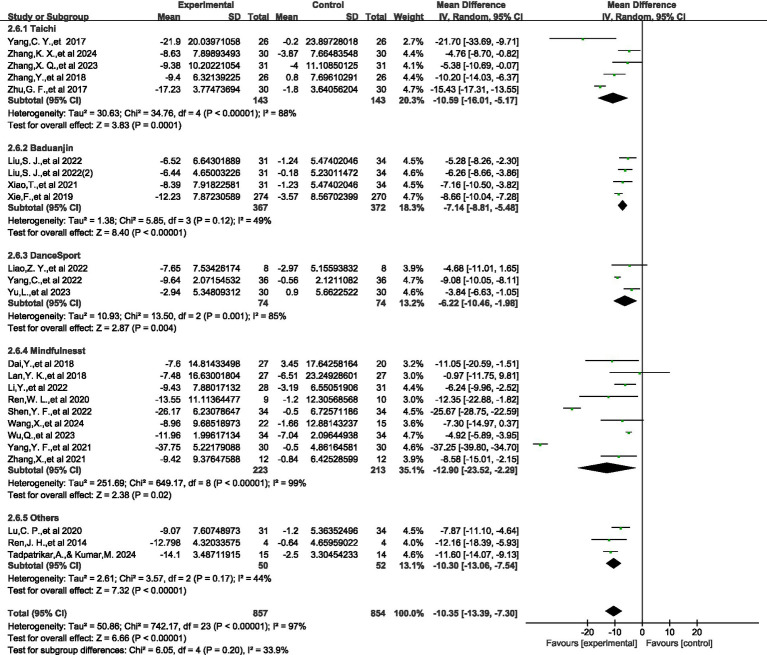
The impact of different physical and mental exercises on the improvement of symptoms of IAD.

**Figure 6:**
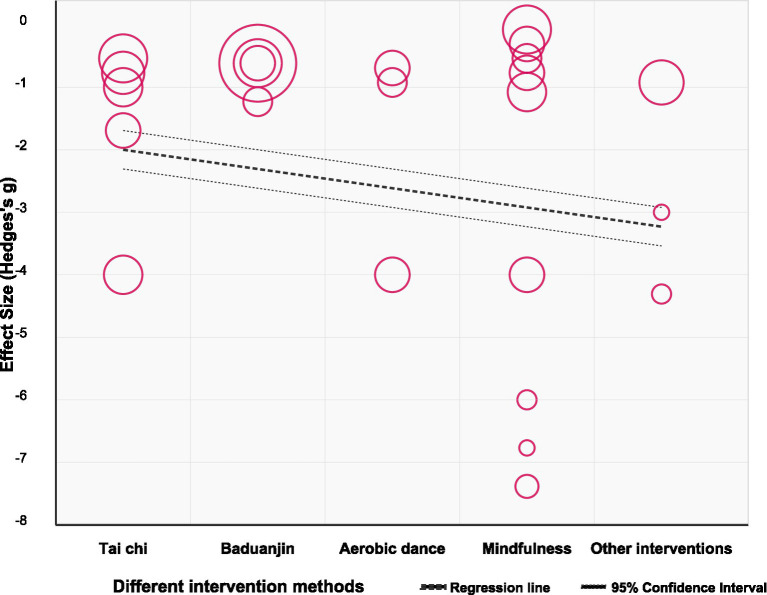
Regression bubble chart.

Figure 5 shows the impact of different physical and mental exercises on young Adults IAD test scores. We screened all included studies and conducted screening based on the original implementation status. In the study, we divided the data into Tai Chi (*n* = 5), Baduanjin (*n* = 4), aerobic fitness exercises (*n* = 3), mindfulness meditation (*n* = 9), and other intervention methods (*n* = 3). Compared with the control group, the intervention group had a significant positive effect on the scores of young Adults IAD tests [SMD = −10.35, 95% CI (−13.39, −7.30), *p* < 0.0001], indicating that the degree of IAD in the intervention group was significantly reduced. The physical and mental exercise intervention method involved in the experiment had a strong positive effect on IAD and the effect was significantly better than that of the control group. However, there is a high degree of heterogeneity among the studies (*I*^2^ = 92%). Subgroup analysis showed that although the differences between subgroups did not reach statistical significance (*p* = 0.23), there were apparent differences in the effect size values: mindfulness meditation [SMD = −12.90, 95% CI (−23.52, −2.29)] and Tai Chi [SMD = −10.59, 95% CI (−16.01, −5.17)] showed higher effect sizes, which is consistent with the results shown in Figure 6. However, the notably wide confidence intervals for these subgroups suggest substantial statistical instability in these estimates, warranting caution in interpretation. These wide intervals likely reflect the limited number of studies in each subgroup and considerable methodological variation.

Although the effect of Ba Duan Jin [SMD = −7.14, 95% CI (−8.81, −5.48)] is relatively small, it has a narrow confidence interval and low heterogeneity (*I*^2^ = 49%), providing the most accurate and reliable estimate. Given the instability of some subgroup findings, these comparative effectiveness results should be considered preliminary rather than definitive, and future research with larger samples is needed to confirm potential differential effects between mind–body exercise modalities.

### Subgroup analysis

The study conducted subgroup analysis based on “exercise frequency, exercise time, intervention cycle, measurement tools, and intervention intensity.” The analysis results are shown in Table 2.

According to Tables 3, 4, there is significant statistical heterogeneity among the 24 studies included [*I*^2^ = 92%, *Q* = 273.91, *p* < 0.0001]. Notwithstanding this substantial heterogeneity, the aggregate effect size [SMD = −1.63, 95% CI (−2.04, −1.22)] demonstrates a statistically significant therapeutic effect of mind–body exercise interventions on young adults’ Internet Addiction Disorder symptomatology.

**Table 3:** Subgroup analysis to assess the effect of interventions on young Adults’ intervention addiction.

Variable	Number of trials	Sample size		Test for overall effect		Heterogeneity			Difference between group *p*
		EG	CG	*Z*	*p*	*I*^2^ (%)	*Q*	*p*	
All	24	857	854	5.77	<0.00001	92	273.91	<0.00001	-
Different intervention cycles									
≦10	15	611	596	5.77	<0.00001	94	215.47	<0.00001	0.48
>10	9	246	258	5.12	<0.00001	86	57.89	<0.00001	
Different exercise frequencies									
Low (≦2times/week)									0.94
	10	259	262	4.00	<0.0001	93	136.23	<0.00001	
Medium (=3times/week)									
	9	227	233	4.18	<0.0001	92	103.03	<0.00001	
High (>3times/week)									
	5	371	359	4.45	<0.00001	87	31.42	<0.00001	
Different exercise times									
Short (≦60 min/time)									0.47
	12	590	588	5.65	<0.00001	93	153.05	<0.00001	
Medium (60–90 min/time)									
	8	178	181	4.22	<0.0001	84	45.13	<0.00001	
Long (>90 min/time)									
	4	89	85	2.43	=0.01	96	74.15	<0.00001	
Different measuring tools									
MPAI	10	501	499	5.69	<0.00001	85	60.60	<0.00001	0.28
MPATS	3	89	95	2.46	=0.01	95	38.18	<0.00001	
Others	11	267	260	4.35	<0.0001	94	171.12	<0.00001	
Single week exercise dose									
High (>1,000 mg)									0.46
	9	217	223	4.52	<0.00001	85	54.18	<0.00001	
Middle (300–999 mg)									
	7	443	437	4.98	<0.00001	93	82.66	<0.00001	
Low (<300 mg)									
	8	197	194	3.33	0.0009	95	135.39	<0.00001	

EG, Experimental group; CG, Control group; MPAI, Mobile Phone Addiction Index (Leung); MPATS, Mobile Phone Addiction Tendency Scale for College Students (Xiong); Others, CIAS, Chinese Internet Addiction Scale (Chen); IAT, Internet Addiction Test (Young); SAS-SV, Smartphone Addiction Scale-Short Version; SCL-90, Symptom Checklist-90 (Young’s Internet Addiction Related Psychological Scale); SAS-C, The Smartphone Addiction Scale for College Students (Su Shuang); SAS-CA, Adult Smartphone Addiction Scale (Chen Huan); PVGUA, Pathological Online Game Usage Questionnaire; Pa value for the between-subgroup difference; Pb value for the heterogeneity within subgroups by Q test.

**Table 4:** Standardized mean differences for each subgroup.

Subgroups		SMD [95%CI]
Different intervention cycles	≦10	−1.75 [−2.34, −1.15]
	>10	−1.45 [−2.01, −0.90]
	ALL	−1.63 [−2.04, −1.22]
Different exercise frequencies	Low (≤2 times/week)	−1.62 [−2.42, −0.83]
	Medium (3 times/week)	−1.75 [−2.56, −0.93]
	High (>3 times/week)	−1.55 [−2.23, −0.87]
	ALL	−1.63 [−2.04, −1.22]
Different exercise times	Short (≤60 min/time)	−1.59 [−2.14, −1.04]
	Medium (60–90 min/time)	−1.34 [−1.96, −10.72]
	Long (>90 min/time)	−2.71 [−4.89, −0.53]
	ALL	−1.63 [−2.04, −1.22]
Different measuring tools	MPAI	−1.26 [−1.69, −0.82]
	MPATS	−2.00 [−3.60, −0.41]
	Others	−1.98 [−2.87, −1.09]
	ALL	−1.63 [−2.04, −1.22]
Single week exercise dose	High (>1,000 mg)	−1.33 [−1.90, −0.75]
	Middle (300–999 mg)	−1.87 [−2.61, −1.14]
	Low (<300 mg)	−1.82 [−2.90, −0.75]
	ALL	−1.63 [−2.04, −1.22]

Analysis of different intervention cycles shows that both short intervention cycles (<10 times) and long intervention cycles (≥10 times) have significant intervention effects on young Adults IAD through physical and mental exercise (both *p* < 0.0001). Both subgroups showed high heterogeneity, with higher heterogeneity observed in the short intervention period (*I*^2^ = 94%, *Q* = 215.47) compared to the short intervention period (*I*^2^ = 86%, *Q* = 57.89). Analysis of different exercise frequencies showed that low frequency (≤2 times/week), medium frequency (=3 times/week), and high frequency (>3 times/week) all produced statistically significant effects (all *p* < 0.00001). Analysis of different exercise durations revealed that extended session interventions (>90 min/session) demonstrated the most pronounced effect magnitude (SMD = −2.71), albeit with concomitant elevation in inter-study variability as evidenced by heterogeneity indices (*I*^2^ = 96%, *Q* = 74.15). Analysis of different measurement tools shows that studies using MPATS measurement exhibit high strain efficiency (SMD = −2.00) and moderate heterogeneity (*I*^2^ = 95%, *Q* = 38.18). Single week exercise dose analysis showed that different dose levels produced significant intervention effects (all *p* < 0.00001), but the low-dose group had higher heterogeneity (*I*^2^ = 95%, *Q* = 135.39).

Despite comprehensive stratified analyses, the substantial heterogeneity between studies (*I*^2^ = 92%) remained predominantly unexplained, a phenomenon frequently encountered in meta-analytic evaluations of complex behavioral interventions (52). This heterogeneity may be attributable to unmeasured moderating variables, methodological variations in intervention implementation protocols, and research design discrepancies (53). As articulated by Guyatt et al., when intervention effects demonstrate consistent directionality and statistical significance across studies, therapeutic efficacy may be reasonably inferred despite the presence of heterogeneity (53). It warrants emphasis that all subgroup analyses revealed statistically significant therapeutic effects of mind–body exercise on IAD symptomatology (all *p* < 0.00001), suggesting that despite methodological and clinical heterogeneity, the intervention efficacy remains robust across diverse experimental conditions.

### Sensitivity analysis

**Figure 7:**
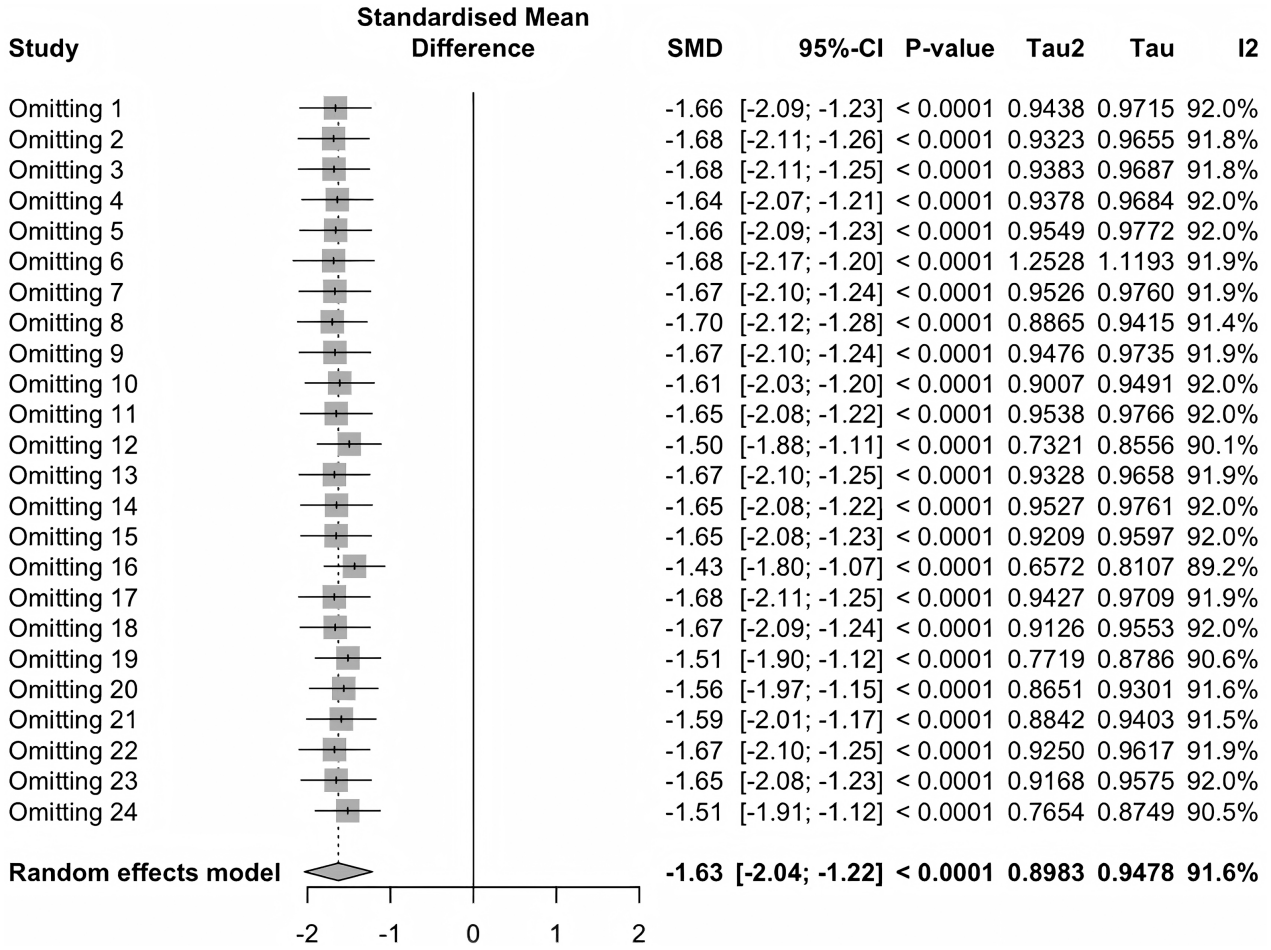
Sensitivity analysis.

Sensitivity analysis showed that regardless of which study was excluded (Figure 7), the standardized mean deviation (SMD) remained between −1.43 and −1.70, with *p* values < 0.0001, indicating consistent statistical significance.

### Publication bias

**Figure 8:**
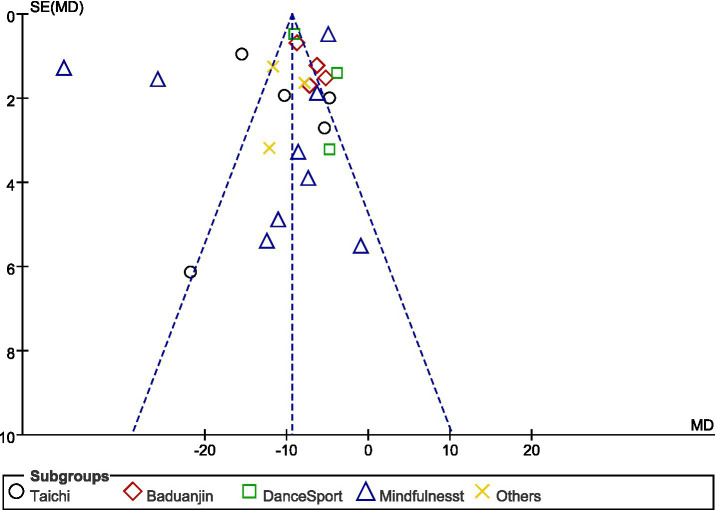
Adjusted comparison funnel plot.

Examination of the funnel plot (Figure 8) suggests potential publication bias in our meta-analysis. The distribution of studies shows some asymmetry, with several small studies (having larger standard errors) displaying more negative effect sizes than would be expected in an unbiased sample. Studies are clustered predominantly on the left side of the mean effect size (represented by the vertical dotted line), particularly those with larger standard errors. This pattern indicates that smaller studies with less significant or positive results might be underrepresented in the literature. However, most studies fall within the expected triangular region defined by the funnel boundaries, suggesting that the observed asymmetry may not severely compromise the validity of our findings. The overall effect size (SMD = −1.63, 95% CI: −2.04, −1.22) appears robust, as demonstrated in our forest plot analysis (Figure 3), with consistent negative effect directions across the 24 included studies. While some publication bias cannot be ruled out, the magnitude and consistency of effects across multiple studies, particularly those with larger sample sizes, support the reliability of our main conclusion that the intervention significantly reduces the primary outcome measure compared to control conditions.

### Bayesian dose-response meta-analysis results

#### The overall dose-response relationship of exercise

**Figure 9:**
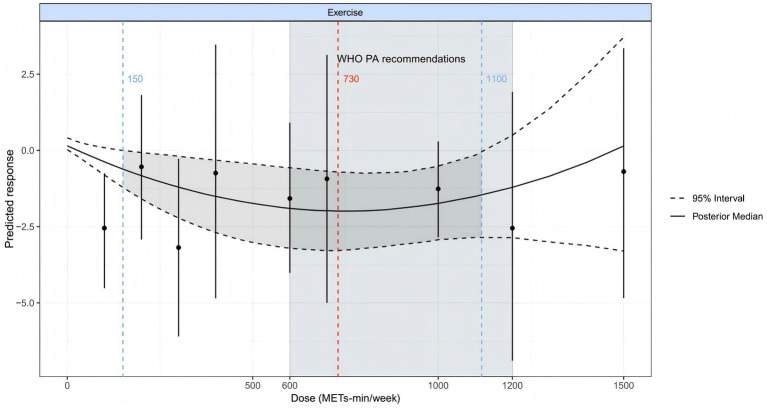
The dose-response relationship of improving IAD through physical and mental exercise. The gray area represents statistically significant regions (*p* < 0.05), where the confidence interval does not cross zero effects, indicating reliable improvement in internet addiction. The red dashed line represents the optimal dosage for achieving the best results, while the black short solid line represents the original dataset. MET, task metabolic equivalent; PA, Physical activity; SMD, Standardized mean deviation; WHO, World Health Organization.

The overall dose-response relationship between exercise dosage and internet addiction exhibits a non-linear dose-dependent U-shaped curve (Figure 9). When exercise dosage is below the threshold of 150 MET min/week (blue dashed line on the left), therapeutic efficacy for IAD symptomatology demonstrates limited magnitude with substantial response variability; as dosage parameters increase, therapeutic efficacy exhibits progressive enhancement, reaching optimal efficacy at 730 MET min/week; upon exceeding the threshold of 1,100 MET min/week (blue dashed line on the right), marginal therapeutic benefit demonstrates significant attenuation, with concomitant expansion of the 95% confidence interval (dashed boundary), indicating increased outcome uncertainty in high-dosage regimens. At a weekly level of 600MET [the recommended lower limit of physical activity for energy expenditure by the World Health Organization (54)], the predictive effect was high (SMD = −1.92, 95% Crl −3.22, −0.58, SD = 0.67). Similarly, at 1200 METmin/week (the recommended upper limit for energy expenditure and physical activity by the World Health Organization), the predictive effect is moderate (SMD = −1.20, 95% Crl −2.86, 0.49, SD = 0.86), but the SD value is larger. The optimal intervention effect was achieved at 730MET per week (SMD = −1.99, 95% CrI −3.28, −0.71, SD = 0.65).

## Discussion

### Main findings

This study analyzed the impact of physical and mental exercise on young Adults IAD and its dose-response relationship. The seven mind–body exercise interventions examined demonstrated statistically significant therapeutic efficacy for IAD symptomatology. Intervention-specific subgroup analyses revealed differential therapeutic potency across modalities, with Mindfulness [SMD = −12.90, 95% CI (−23.52, −2.29)] demonstrating the most pronounced aggregate effect magnitude, followed by Tai Chi [SMD = −10.59, 95% CI (−16.01, −5.17)], other intervention methodologies [SMD = −10.30, 95% CI (−13.06, −7.54)], Baduanjin [SMD = −7.14, 95% CI (−8.81, −5.48)], and Aerobicdance [SMD = −6.22, 95% CI (−10.46, −1.98)]. It warrants acknowledgment that the expansive confidence intervals observed for certain intervention modalities suggest substantial statistical imprecision in these effect estimations.

The study also found a non-linear dose-response relationship between physical and mental exercise and IAD, exhibiting a typical U-shaped relationship. The estimated optimal effective dose is 730 MET min/week, which is equivalent to 221 min of Qigong (3.3 MET), 152 min of DanceSports (4.8 MET), 317 min of Yoga (2.3 MET), 730 min of Mindfulness (1.0 MET), 122 min of Tai chi (6.0 MET), 221 min of Baduanjin (3.3 MET), or 209 min of Aerobicdance (3.5 MET). When the physical and mental exercise dose reaches 1,100 MET minutes per week, the improvement of IAD symptoms reaches a stable state. It is worth noting that the optimal dose of 730 MET min/week observed by us falls within the recommended range of physical activity by the World Health Organization (WHO) (600–1,100 MET min/week, light blue area in the figure), indicating that following WHO’s general activity recommendations is also applicable for improving symptoms of IAD and does not require special adjustments. Finally, sensitivity analysis showed that factors such as high risk of bias, short intervention time, and different measurement tools did not significantly affect the effectiveness of physical and mental exercise interventions.

### Characteristics and limitations of included RCTs

#### Study characteristics and methodological heterogeneity

The 24 included randomized controlled trials, spanning from 2000 to 2025, exhibited substantial methodological heterogeneity that requires careful consideration. The studies involved 1,711 young adults aged 18–24 years with a median age of 24, representing a relatively homogeneous population in terms of age demographics. However, several methodological variations were observed across studies that may influence the interpretation of our findings.

Intervention characteristics and variability: The intervention protocols varied considerably across studies. Intervention duration ranged from 4 weeks to 16 weeks (median: 10 weeks), with frequency varying from 1 to 10 times per week (median: 5.5 times), and session duration spanning 30–150 min (median: 90 min). This wide variation in dosage parameters created challenges for standardization and may contribute to the observed heterogeneity (*I*^2^ = 92%). The diverse implementation approaches across different cultural contexts (predominantly Chinese studies with one Indian study) may also introduce cultural and methodological variations that affect intervention effectiveness.

Measurement tool inconsistencies: A significant methodological challenge emerged from the use of multiple IAD assessment instruments across studies. The included studies employed various scales including MPAI, MPATS, CIAS, IAT, SAS-SV, SCL-90, SAS-C, SAS-CA, and PVGUA. While all these instruments measure aspects of internet-related addiction behaviors, they differ in their focus areas (general internet addiction vs. smartphone addiction vs. gaming addiction), scoring systems, and psychometric properties. This heterogeneity in outcome measurement tools may have contributed to the observed statistical heterogeneity and complicates direct comparison across studies.

#### Risk of bias assessment and quality concerns

The risk of bias assessment revealed significant methodological limitations across the included studies. Only 3 studies (12.5%) were assessed as low-risk, while 11 studies (45.8%) had certain methodological concerns, and 10 studies (41.7%) showed high-risk bias. The most prominent issue was implementation bias, with approximately 95% of studies showing high risk regarding participant and researcher blinding. This limitation is inherent to physical and mental exercise intervention research, as complete blinding of intervention types is often practically impossible.

Randomization and allocation concerns: Approximately 50% of studies showed unclear risk in random sequence generation and allocation concealment, primarily due to insufficient methodological details in the original research reports rather than fundamental design flaws. This lack of transparency in reporting randomization procedures raises concerns about selection bias and the overall validity of treatment effect estimates.

Data integrity and reporting quality: Encouragingly, the risk of incomplete outcome data (attrition bias) and selective reporting (reporting bias) was relatively low, with approximately 70–80% of studies rated as low-risk. This suggests reasonable methodological quality in terms of data integrity and reporting transparency, which supports the reliability of the primary outcome data used in our meta-analysis.

#### Methodological challenges identified through systematic review

Sample size limitations: Several studies included in our analysis had relatively small sample sizes, with some interventions represented by very few participants (e.g., 4 participants for sports dance, 15 for yoga). These small sample sizes limit the precision of effect estimates and may contribute to the wide confidence intervals observed in some subgroup analyses.

Control group heterogeneity: The control conditions varied across studies, ranging from waitlist controls to regular physical activity groups. This variation in control group characteristics may influence the magnitude of observed treatment effects and contribute to between-study heterogeneity.

Cultural and contextual factors: The predominance of studies conducted in Chinese populations may limit the generalizability of findings to other cultural contexts. Cultural factors may influence both the acceptability and effectiveness of different mind–body interventions, suggesting that our findings may require validation in more diverse populations.

Comparison of the effects and mechanism explanation of different physical and mental exercise intervention methods.

The research results indicate that mindfulness meditation has a significantly higher effect on improving IAD compared to other physical and mental exercise interventions [SMD = −12.90, 95%CI (−23.52, −2.29), *p* < 0.0001]. While this substantial effect magnitude warrants judicious interpretation given the expansive confidence interval, mindfulness meditation’s potential therapeutic superiority may be attributable to multifaceted neurobiological mechanisms. From a neuroplasticity perspective, Tang et al. demonstrated that mindfulness training significantly potentiates functional connectivity between default mode and executive control networks, thereby optimizing attentional regulation and self-referential processing capacities (55); Hölzel et al. further elucidated that 8-week mindfulness training protocols induce significant volumetric enhancement in prefrontal cortical and anterior cingulate cortical regions (56), structural modifications that directly augment cognitive control capacities and may contribute to executive function enhancement in IAD patients. At the neurotransmitter level, Khoury et al.’s systematic review delineated several neurochemical regulatory pathways associated with mindfulness practice: enhancement of affective state through serotonergic stabilization, anxiolytic effects via GABAergic potentiation, and attenuation of chronic stress responsivity through inhibition of hypothalamic–pituitary–adrenal axis activation and subsequent cortisol secretion (57). Regarding social cognition, Vago and Silbersweig’s investigation demonstrated that mindfulness practice significantly enhances participants’ self-awareness and interpersonal functioning through insular activation and potentiation of functional connectivity between anterior cingulate cortical and prefrontal regions (58).

In contrast, traditional Chinese sports such as Tai Chi and Baduanjin have also played a positive role in improving IAD. Tai Chi combines the characteristics of physical activity, breath control, and mindfulness, and research has shown that it can effectively regulate the function of the autonomic nervous system. The review by Song et al. suggests that practicing Tai Chi can increase vagus nerve activity, reduce sympathetic nervous system excitability, and thus lower levels of stress hormones such as cortisol. This has positive implications for alleviating the stress response hyperactivity commonly found in internet addicts (59). Wang et al. confirmed through a randomized controlled trial that 24 weeks of Tai Chi practice can significantly reduce anxiety levels in college students (*p* < 0.01) and improve heart rate variability indicators, indicating positive regulation of autonomic nervous system function. In terms of brain function (60), Wei et al. used functional magnetic resonance imaging technology to find that after 12 weeks of Tai Chi training, participants showed increased activity in the prefrontal cortex, especially the dorsolateral prefrontal cortex, which is closely related to executive control and impulse suppression, and executive dysfunction is a typical feature of internet addicts (61). As a traditional Chinese fitness qigong, although the movements of Baduanjin are relatively simple, its impact on the neuroendocrine system is also worth paying attention to. The systematic review by Zou et al. showed that practicing Baduanjin can effectively reduce serum cortisol and norepinephrine levels, while improving mental health (62).

The subgroups composed of Qigong, DanceSports, and Yoga also showed higher efficacy, although their mechanisms of action were different, they have been widely proven to improve IAD. This type of physical and mental activity can effectively regulate the hypothalamic pituitary adrenal (HPA) axis function and improve the common neuroendocrine imbalance in internet addicts. The systematic review by Pascoe et al. confirms that yoga practice can significantly reduce cortisol levels, which is directly related to stress response dysregulation in internet addicts (63). Secondly, these activities optimize the balance of neurotransmitters. Streeter et al. found through magnetic resonance spectroscopy that yoga practice can significantly increase GABA levels, and low GABA levels are closely related to difficulty in impulse control (64). Field et al. found that regular yoga practice can reduce sympathetic nervous system activity while increasing levels of anti stress neurotransmitters (65). In addition, these activities promote the optimization of functional connectivity in key brain regions. The meta-analysis by Fox et al. found that yoga and meditation practices can affect the structure of brain regions related to attention control and emotion regulation, which is crucial for improving self-control related to IAD (66). The study by Li et al. on individuals addicted to online games showed that physical and mental intervention activities can significantly improve their attention control ability and impulse suppression function (67). Chen et al. found that psychological intervention combined with physical activity can effectively reduce internet usage time and improve self-regulation ability (68). The study by Du et al. confirmed that physical activity intervention can significantly improve the internet usage behavior and mental health status of college students (69).

Aerobicdance affect IAD symptoms by regulating various neurotransmitters. Research has shown that aerobic exercise promotes dopamine secretion, which is transmitted through the basal ganglia to the prefrontal cortex and motor cortex, helping to restore the neurological function of addicts (70). This exercise can increase dopamine levels in the striatum, hypothalamus, and brainstem of different genders. Research has found that young Adults with IAD who engage in regular aerobic exercise have significantly higher levels of serum brain-derived neurotrophic factor (BDNF) compared to the non exercise group (*p* < 0.05), and their IAD scores are significantly reduced, with a negative correlation between the two (*r* = −0.58) (71). High intensity exercise stimulates BDNF gene expression through β-hydroxybutyrate ketone bodies, promotes neural plasticity, and improves cognitive function (72). Although Tai Chi, Baduanjin, and Aerobicdance perform well in regulating the autonomic nervous system, improving heart rate variability, and enhancing prefrontal cortex activity, their targeted effects on core attention deficit and emotional control ability in internet addicts are usually not as significant as mindfulness meditation (73), resulting in slightly lower overall intervention effects on IAD symptoms.

These findings not only reveal the underlying mechanisms by which different forms of exercise improve internet addiction, but also have significant implications for public health and clinical practice. Internet addiction is closely related to attention deficit, impulse control disorder, difficulty regulating emotions, and social dysfunction (74). Mindfulness provides a comprehensive intervention strategy for these issues by simultaneously enhancing neural activity in the anterior cingulate gyrus and insula regions and regulating key neurotransmitter systems. For high-risk IAD populations such as teenagers, college students, and professionals facing high stress, Mindfulness—a low threshold, high compliance intervention with dual cognitive and emotional regulation benefits—has considerable potential for application (75, 76) Incorporating mindfulness into campus mental health programs, community health promotion programs, and IAD prevention strategies may provide an economical and feasible approach to improving IAD symptoms, enhancing self-regulation abilities, and reducing related mental health risks.

In summary, Mindfulness as a single intervention method has more high-quality research support (77), and its mechanism in improving attention control, emotion regulation, and impulse control is more clear (56, 78). Mindfulness has shown significant therapeutic effects on IAD by enhancing self-awareness, reducing automated response patterns, and improving executive function. These findings are consistent with the views of Kuss and Lopez Fernandez that interventions based on cognitive and behavioral dual regulation are more effective in alleviating IAD behavior, providing profound guidance for public health policies and clinical practice.

### Dose-response relationship analysis

Research has found a U-shaped relationship between physical and mental exercise and symptoms of IAD, with the optimal physical and mental exercise dose being 730 MET min/week. Li et al. found that among American adults, after 8 weeks of mindfulness guided recovery enhancement (MORE) intervention, participants’ symptoms of online gaming disorder were significantly reduced, especially those who maintained near 750 MET min/week showed the best symptom improvement and maintenance effects (67). Yao et al. reported that a moderate intensity intervention (approximately 750 MET min/week) combining reality therapy and mindfulness can significantly reduce cross temporal decision-making impulsivity in young people with online gaming disorders (79), while interventions below 600 or above 1,000 MET min/week have relatively weaker effects. Low exercise doses cannot fully activate the autonomic mechanism, while high exercise doses may lead to decreased compliance and excessive fatigue. Based on these findings, it is recommended that physical and mental exercise interventions for young people with IAD, including Qigong, DanceSports, Yoga, Mindfulness, Tai chi, and Aerobicdance, should be maintained at the optimal dose of 730 MET min/week to achieve the most ideal intervention effect.

### Practical implications of the U-shaped dose-response relationship for exercise recommendations

Our dose-effect analysis revealed a distinct U-shaped curve relationship between physical-mental exercise and Internet Addiction Disorder (IAD) improvement. Research data identified an optimal exercise intensity point (approximately 730 MET minutes weekly total), where intervention effectiveness reached its peak. This finding holds significant practical value, demonstrating that insufficient or excessive exercise levels both diminish the efficacy of IAD interventions. These results provide scientific basis for developing precise treatment protocols while highlighting the crucial role of moderate exercise in the therapeutic process. On the one hand, insufficient exercise cannot provide sufficient neurobiological stimulation, leading to impaired regulation mechanisms of neurotransmitters such as dopamine and serotonin, which cannot effectively improve symptoms of IAD (80). On the contrary, excessive exercise can cause physical fatigue and increased stress, leading to a decrease in self-regulation ability and exacerbating symptoms of IAD (81). Therefore, only moderate physical and mental exercise can effectively regulate the brain’s reward pathway (82), repair dopamine pathway blockages caused by IAD, and become a key factor in improving IAD.

This U-shaped correlation additionally establishes a foundation for tailored exercise regimens. The quantification of physical activity through standardized metrics like MET minutes enables healthcare practitioners and public health specialists to precisely determine optimal exercise parameters—including intensity levels and time commitments—for maximizing therapeutic outcomes in IAD patients. Such evidence-based recommendations allow for individualized treatment approaches that can effectively target IAD symptomatology through carefully calibrated physical activity protocols. For example, for young people who have already shown a tendency toward IAD, adhering to this optimal exercise dosage can effectively activate the normal regulatory mechanisms of neurotransmitters such as dopamine and serotonin, help restore prefrontal cortex function, enhance executive control ability, and avoid negative effects associated with excessive exercise.

In addition, this finding is consistent with the guidelines for moderate intensity physical and mental exercise intervention recommended by the World Health Organization (54) and the IAD Research Association (83), which further emphasizes the importance of maintaining an appropriate level of physical activity when dealing with IAD. Incorporating this optimal exercise dosage into campus health education, psychological counseling services, and young Adults health promotion programs can not only effectively alleviate symptoms of IAD, but also improve mental health problems such as depression, anxiety, and social disorders caused by excessive internet use (67), thereby promoting the physical and mental health development of young Adults on a broader scale.

In summary, the U-shaped dose-response relationship revealed in this study suggests that precise regulation of exercise dosage is crucial in developing physical and mental exercise interventions aimed at alleviating symptoms of IAD. By adjusting the intensity and duration of exercise appropriately to achieve the optimal dosage, symptoms of IAD can be minimized to the greatest extent possible, and executive function and self-regulation abilities can be improved. This discovery provides clear clinical guidance for exercise prescriptions targeting young people with IAD, and has substantial application value for educational institutions, mental health services, and public health policy-making. Table 5 lists exercise recommendations to improve symptoms of IAD.

**Table 5:** Physical and mental exercise recommendations for improving symptoms of IAD.

Type	Energy expenditure^a^ (METs-min)	Minimum recommended accumulation^b^ (min/week)	Minimum recommendations for exercise prescription^c^ (sessions × min/per week)
Mindfulness	1.0 (code 07075)	~730	~7 × 105
Tai Chi	6.0 (code 15674)	~122	~3 × 40
Qigong	3.3 (code 15670)	~221	~5 × 45
DanceSports	4.8 (code 02005)	~152	~3 × 50
Yoga	2.3 (code 02175)	~317	~5 × 65
Baduanjin	3.3 (code 15670)	~221	~5 × 45
Aerobicdance	3.5 (code 02030)	~209	~4 × 50

a Intensity coding was extracted from the 《Compendium of Physical Activities》.

b Minimum weekly time of exercise.

c Frequency and duration of each exercise, not counting warm-up and cool-down.

### Comparison with existing studies

The results of this study are highly consistent with existing literature. Previous investigations have demonstrated that low to moderate intensity mind–body interventions produce statistically significant therapeutic effects in ameliorating IAD symptomatology (84). Gong and Lin’s empirical research corroborates that moderate to low-intensity mindfulness meditation protocols effectively enhance psychological wellbeing among collegiate populations, including reduction of anxiety and depressive symptomatology frequently comorbid with IAD. These findings provide additional theoretical substantiation for the efficacy of mindfulness in attenuating IAD-related psychopathology.

In contradistinction to conventional meta-analytic methodologies, this investigation employed comparative effect measure quantification to systematically evaluate and hierarchically categorize the relative efficacy of diverse intervention modalities. Furthermore, this study represents the first implementation of Bayesian dose-response analytical techniques to empirically quantify optimal dosage parameters for mind–body exercise interventions in IAD, thereby establishing an evidence-based foundation for the development of personalized intervention protocols.

### Practical and applied significance

The empirical findings herein demonstrate substantial therapeutic efficacy for non-pharmacological intervention modalities in ameliorating Internet Addiction Disorder symptomatology among young adult populations. Mindfulness-based practices emerge as potentially superior therapeutic modalities for exercise-mediated IAD symptom reduction, though with acknowledgment of statistical limitations. Of particular clinical relevance, adherence to empirically derived optimal exercise dosage parameters (730 MET minutes/week) appears to yield significant enhancement of intervention efficacy. This investigation contributes substantive empirical evidence supporting the implementation of structured physical and contemplative exercise protocols within educational institutions and public health frameworks addressing IAD prevention and treatment among young adult demographic cohorts.

### Methodological strengths and limitations

This investigation’s principal methodological strength resides in its integration of network meta-analytic techniques with Bayesian statistical frameworks, facilitating systematic comparative evaluation of diverse mind–body exercise interventions for young adult IAD symptomatology while simultaneously quantifying optimal dosage parameters. This methodological approach enables synthesis of both direct and indirect comparative evidence, thereby providing more comprehensive efficacy evaluations—a particularly valuable contribution given that previous empirical investigations rarely conducted comparative analyses of multiple intervention modalities within single experimental designs.

Notwithstanding the substantive empirical findings, methodological limitations merit critical examination. Substantial methodological heterogeneity characterizes the corpus of incorporated randomized controlled trials, manifesting in divergent randomization protocols, variable allocation concealment procedures, inconsistent blinding methodologies, and heterogeneous outcome reporting mechanisms. Studies exhibiting elevated or indeterminate bias coefficients potentially attenuate effect size estimation precision and circumscribe the generalizability of observed therapeutic efficacy parameters.

Furthermore, analyses predominantly relied on self-reported IAD measurements (MPAI, MPATS, SAS-SV, etc.). These instruments are susceptible to recall bias and social desirability effects, potentially compromising outcome accuracy. Inconsistent questionnaire item interpretation across studies may introduce additional variability in IAD assessment metrics.

## Conclusion

Bayesian network meta-analysis in this investigation confirmed significant IAD improvement effects from various physical and mental exercise modalities among young adults, with mindfulness demonstrating particularly robust efficacy. Analysis revealed a distinct U-shaped dose-response correlation between exercise interventions and IAD symptom amelioration. The identified optimal intervention dosage approximates 730 MET minutes weekly. Exercise regimens below 150 MET minutes weekly yield limited benefits with substantial variability, while dosages exceeding 1,100 MET minutes weekly exhibit diminishing marginal returns. Results underscore the critical importance of moderate activity levels for IAD improvement, as excessive exercise potentially diminishes intervention effectiveness through induced fatigue and physiological stress responses. These findings provide valuable guidance for developing evidence-based exercise intervention protocols, optimizing methodological approaches and dosage parameters, and elucidating underlying mechanisms through which physical-mental exercise mitigates IAD symptomatology in subsequent research initiatives.

## Data Availability

The original contributions presented in the study are included in the article/Supplementary material, further inquiries can be directed to the corresponding authors.

## References

[1] Shaw M Black DW. Internet addiction: definition, assessment, epidemiology and clinical management. CNS Drugs. (2008) 22:353–65. doi: 10.2165/00023210-200822050-00001, PMID: 18399706

[2] Manus J-M. DSM-5-TR diagnostic and statistical manual of mental disorders. Revue Francophone Des Laboratoires. (2024) 2024:14. doi: 10.1016/s1773-035x(24)00069-8

[3] Meng SQ Cheng JL Li YY Yang XQ Zheng JW Chang XW . Global prevalence of digital addiction in general population: a systematic review and meta-analysis. Clin Psychol Rev. (2022) 92:102128. doi: 10.1016/j.cpr.2022.102128, PMID: 35150965

[4] Stockdale L Coyne SM. Video game addiction in emerging adulthood: cross-sectional evidence of pathology in video game addicts as compared to matched healthy controls. J Affect Disord. (2018) 225:265–72. doi: 10.1016/j.jad.2017.08.045, PMID: 28841491

[5] Takeuchi H Taki Y Hashizume H Asano K Asano M Sassa Y . Impact of videogame play on the brain's microstructural properties: cross-sectional and longitudinal analyses. Mol Psychiatry. (2016) 21:1781–9. doi: 10.1038/mp.2015.193, PMID: 26728566 PMC5116480

[6] Hossin MZ Islam A Billah M Haque M Uddin J. Is there a gradient in the association between internet addiction and health? PLoS One. (2022) 17:e0264716. doi: 10.1371/journal.pone.0264716, PMID: 35239733 PMC8893621

[7] Chang CH Chang YC Yang L Tzang R-F. The comparative efficacy of treatments for children and young adults with internet addiction/internet gaming disorder: an updated meta-analysis. Int J Environ Res Public Health. (2022) 19:2612. doi: 10.3390/ijerph19052612, PMID: 35270305 PMC8909504

[8] Király O Potenza MN Stein DJ King DL Hodgins DC Saunders JB . Preventing problematic internet use during the COVID-19 pandemic: consensus guidance. Compr Psychiatry. (2020) 100:152180. doi: 10.1016/j.comppsych.2020.152180, PMID: 32422427 PMC7215166

[9] Zhu S Zhuang Y Lee P Li JC-M Wong PWC. Leisure and problem gaming behaviors among children and adolescents during school closures caused by COVID-19 in Hong Kong: quantitative cross-sectional survey study. JMIR Serious Games. (2021) 9:e26808. doi: 10.2196/26808, PMID: 33960954 PMC8108935

[10] Throuvala MA Griffiths MD Rennoldson M Kuss DJ. School-based prevention for adolescent internet addiction: prevention is the key. A systematic literature review. Curr Neuropharmacol. (2019) 17:507–25. doi: 10.2174/1570159X16666180813153806, PMID: 30101714 PMC6712298

[11] Kuss DJ Lopez-Fernandez O. Internet addiction and problematic internet use: a systematic review of clinical research. World J Psychiatry. (2016) 6:143–76. doi: 10.5498/wjp.v6.i1.143, PMID: 27014605 PMC4804263

[12] King DL Delfabbro P Wu AMS Doh YY Kuss DJ Pallesen S . Treatment of internet gaming disorder: an international systematic review and CONSORT evaluation. Clin Psychol Rev. (2017) 54:123–33. doi: 10.1016/j.cpr.2017.04.002 28458097

[13] Arya S Kaji AH Boermeester MA. PRISMA reporting guidelines for meta-analyses and systematic reviews. JAMA Surg. (2021) 156:789–90. doi: 10.1001/jamasurg.2021.0546 33825806

[14] Higgins J Thomas J Chandler J Cumpston M Li T Page MJ. Cochrane handbook for systematic reviews of interventions. 2nd ed. Chichester (UK): John Wiley & Sons, Ltd (2019).

[15] López-Valenciano A Mayo X Liguori G Copeland R Lamb M Jiménez A. Changes in sedentary behaviour in European Union adults between 2002 and 2017. BMC Public Health. (2020) 20:1–10. doi: 10.1186/s12889-020-09293-1 32843022 PMC7448983

[16] Herrmann SD Willis EA Ainsworth BE Barreira TV Hastert M Kracht CL . 2024 adult compendium of physical activities: a third update of the energy costs of human activities. J Sport Health Sci. (2024) 13:6–12. doi: 10.1016/j.jshs.2023.10.010, PMID: 38242596 PMC10818145

[17] American College of Sports Medicine. ACSM's guidelines for exercise testing and prescription Lippincott Williams & Wilkins (2013). 10.1249/JSR.0b013e31829a68cf 23851406

[18] Wasfy MM Baggish AL. Exercise dose in clinical practice. Circulation. (2016) 133:2297–313. doi: 10.1161/CIRCULATIONAHA.116.018093, PMID: 27267537 PMC4902280

[19] Wang D Zhang P. Meta analysis of the effect of different forms of exercise on the prevention and treatment of carotid atherosclerosis. Sports Sci. (2019) 39:75–87.

[20] Zhang T Dong S Zhou Z. Advanced meta analysis method. Shanghai: Fudan University Press.

[21] Liu Y Li L Ma Y Liang Z Sun Z Cui J . Meta analysis of the incidence of internet addiction among Chinese college students. Chin J Evid Based Med. (2021) 21:61–8.

[22] Wu Y Chen L Yu Z Liu Y. Meta analysis of the effect of exercise on the prevention and treatment of non-alcoholic fatty liver disease. Fujian Sports Sci Technol. (2020) 39:1–8.

[23] Bürkner PC. Advanced Bayesian multilevel modeling with the R package brms. R J. 10:395. doi: 10.32614/RJ-2018-017

[24] Etzioni RD Kadane JB. Bayesian statistical methods in public health and medicine. Annu Rev Public Health. (1995) 16:23–41. doi: 10.1146/annurev.pu.16.050195.000323, PMID: 7639872

[25] Williams D. R. Rast P. Bürkner P. C. Bayesian meta-analysis with weakly informative prior distributions. (2018).

[26] Betancourt M Girolami M. Hamiltonian Monte Carlo for hierarchical models In: Current trends in Bayesian methodology with applications, vol. 79 (2015). 2–4.

[27] Brooks SP Gelman A. General methods for monitoring convergence of iterative simulations. J Comput Graph Stat. (1998) 7:434–55. doi: 10.1080/10618600.1998.10474787

[28] Yang CY Zeng GF. Effects of Tai Chi exercise on internet addiction among college students. Chin J Sch Health. (2017) 38:292–4.

[29] Zhang X Yang H Zhang K Zhang J Xiaoyan L Guo H . Effects of exercise or tai chi on Internet addiction in college students and the potential role of gut microbiota: A randomized controlled trial. J Affect Disord. (2023) 327:404–15. doi: 10.1016/j.jad.2023.02.002 36754096

[30] Xiao T Jiao C Yao J Yang Z Liu S . Effects of basketball and Baduan exercise interventions on problematic smartphone use and mental health among college students: a randomized controlled trial. Evid Based Complement Alternat Med. (2021) 2021:8880716. doi: 10.1155/2021/8880716 33574886 PMC7864751

[31] Zhang K Guo H Zhang X Yang H Yuan G Zhu Z . Effects of aerobic exercise or Tai Chi Chuan interventions on problematic mobile phone use and the potential role of intestinal flora: a multi-arm randomized controlled trial. J Psychiatr Res. (2024) 170:394–407. doi: 10.1016/j.jpsychires.2023.12.032 38218013

[32] Lan Y Ding JE Li W Li J Zhang Y Liu M . A pilot study of a group mindfulness-based cognitive-behavioral intervention for smartphone addiction among university students. J Behav Addict. (2018) 7:1171–6. doi: 10.1556/2006.7.2018.103 30418075 PMC6376383

[33] Ren JH Ao ZH He CL Bo L Jiawei W. Experimental Study on Sports Intervention for Internet Addiction in College Students [J]. Heilongjiang Med Sci. (2014) 37:93–4.

[34] Xie F. Status of mobile phone addiction among nursing students and intervention research using Baduanjin health education Shanxi Medical University (2019).

[35] Zhang Y. Research on the relationship between college students’ mobile phone dependence and physical health and exercise intervention Gannan Normal University (2018).

[36] Liu SJ Liu YN Wang L. An empirical study on exercise intervention and group cognitive therapy for college students’ mobile phone dependence. Chin J Sch Health. (2022) 43:825–9.

[37] Li Y. Effects of mindfulness training on response inhibition in college students with mobile phone addiction Guizhou Medical University (2022).

[38] Lu C Zou L Becker B Mark DG Yu Q Chen S-T . Comparative effectiveness of mind-body exercise versus cognitive behavioral therapy for college students with problematic smartphone use: a randomized controlled trial[J]. Int J Ment Health Promot. (2020) 22:271–82. doi: 10.32604/IJMHP.2020.014419

[39] Yang C. Experimental Study on the Effect of Aerobic Fitness and Badminton on Moderate Cell Phone Addiction in Vocational School Students[D] Yangzhou University (2022) doi: 10.27441/d.cnki.gyzdu.2022.000378

[40] Dai Y. A Randomized Controlled Study of Mindfulness Training to Reduce Mobile Phone Dependence Level in Mobile Phone Dependents[D] Zhejiang Normal University (2018).

[41] Liu SJ. Research on the Intervention Effect of Different Motor Skills and Group Cognition on College Students’ Mobile Phone Dependence[D] Wuhan University of Technology (2019) doi: 10.27381/d.cnki.gwlgu.2019.001621

[42] Zhang X. Study on the Influence and Intervention of Junior Middle School Students’ Perceived Social Support and Loneliness on Mobile Phone Dependence[D] Hebei University (2021) doi: 10.27103/d.cnki.ghebu.2021.001962

[43] Yang YF. Research on the Influence of College Students’ Mobile Phone Psychological Craving on Mobile Phone Addiction and Addiction Intervention[D] Jiangnan University (2021) doi: 10.27169/d.cnki.gwqgu.2021.001830

[44] Yu L Xia JM Lei Y. Research on the comprehensive intervention of aerobic exercise on female college students’ mobile phone addiction[J]. Sichuan Sports Sci. (2023) 42:70–3. doi: 10.13932/j.cnki.sctykx.2023.06.15

[45] Liao ZY Mai ZZF Chen ZJ. Effects of basketball and aerobic exercise intervention on improving smartphone addiction symptoms and mental health of college students[J]. Sports Sci Technol Lit Bull. (2022) 30:160–162,171. doi: 10.19379/j.cnki.issn.1005-0256.2022.06.043

[46] Zhu GF. Research on physical exercise intervention for college students’ mobile phone addiction tendency[J]. Zhejiang Sports Sci. (2017) 39:90–7.

[47] Tadpatrikar A Sharma MK Bhargav H . Yoga as an adjuvant with multimodal psychological interventions for excessive use of technology: a randomized controlled trial from India[J]. Int J Yoga. (2024) 17:37–45. doi: 10.4103/ijoy.ijoy_187_23 38899141 PMC11185431

[48] Wu Q Shen Y Yuan QQ Qiu J Li Y. The impact of mindfulness intervention on adolescent patients with internet game addiction [J]. Huaxia Medical. (2023) 36:169–72. doi: 10.19296/j.cnki.1008-2409.2023-032-32

[49] Wang X Cheng ZH Peng Y Niu C. A Study on the Intervention of Mindfulness Cognitive Therapy in Improving Anxiety and Depression in College Students with Mobile Phone Addiction [J]. Psychology Monthly. (2024) 19:69–73. doi: 10.19738/j.cnki.psy.2024.19.019

[50] Ren WL. The Intervention Effect of Mindfulness Training on High School Students’ Mobile Game Addiction [D] Hebei North University (2020) doi: 10.27767/d.cnki.ghbbf.2020.000192

[51] Shen YF. The Effect of Mindfulness Training on Attentional Bias in College Students with Mobile Phone Dependence[D] North China University of Science and Technology (2022) doi: 10.27108/d.cnki.ghelu.2022.000031

[52] Higgins JPT. Commentary: Heterogeneity in meta-analysis should be expected and appropriately quantified. Int J Epidemiol. (2008) 37:1158–60. doi: 10.1093/ije/dyn204 18832388

[53] Guyatt G Oxman AD Kunz R Woodcock J Brozek J Helfand M . GRADE guidelines: 7. Rating the quality of evidence—inconsistency. J Clin Epidemiol. (2011) 64:1294–302. doi: 10.1016/j.jclinepi.2011.03.017 21803546

[54] Bull F Al-Ansari SS Biddle SJH Bull FC Biddle S Borodulin K . World Health Organization 2020 guidelines on physical activity and sedentary behaviour. Br J Sports Med. (2020) 54:1451–62. doi: 10.1136/bjsports-2020-102955 33239350 PMC7719906

[55] Tang YY Hölzel BK Posner MI. The neuroscience of mindfulness meditation[J]. Nat Rev Neurosci. (2015) 16:213–25. doi: 10.1038/nrn3916 25783612

[56] Hölzel BK Carmody J Vangel M Congleton C Yerramsetti SM Gard T . Mindfulness practice leads to increases in regional brain gray matter density[J]. Psychiatry Res Neuroimaging. (2011) 191:36–43. doi: 10.1016/j.pscychresns.2010.08.006 PMC3004979 21071182

[57] Khoury B Sharma M Rush SE Fournier C. Mindfulness-based stress reduction for healthy individuals: A meta-analysis[J]. J Psychosom Res. (2015) 78:519–28. doi: 10.1016/j.jpsychores.2015.03.009 25818837

[58] Vago DR Silbersweig DA. Self-awareness, self-regulation, and self-transcendence (S-ART): a framework for understanding the neurobiological mechanisms of mindfulness[J]. Front Hum Neurosci. (2012) 6:296. doi: 10.3389/fnhum.2012.00296 23112770 PMC3480633

[59] Song QH Shen GQ Xu RM . Effect of Tai Chi exercise on the physical and mental health of the elder patients suffered from anxiety disorder[J]. Int J Physiol Pathophysiol Pharmacol. (2014) 6:55. 24665359 PMC3961102

[60] Wang C Bannuru RR Ramel J Kupelnick B Scott T Schmid CH. Tai Chi on psychological well-being: systematic review and meta-analysis[J]. BMC Complement Altern Med. (2010) 10:1–16. doi: 10.1186/1472-6882-10-23 20492638 PMC2893078

[61] Wei G-X Xu T Fan F Dong H-M Jiang L Li H-J . Can Taichi reshape the brain? A brain morphometry study[J]. PloS one. (2013) 8:e61038. doi: 10.1371/journal.pone.0061038 23585869 PMC3621760

[62] Zou L Pan Z Yeung A . A review study on the beneficial effects of Baduan**[J]. J Altern Complement Med. (2018) 24:324–35. doi: 10.1089/acm.2017.0241 29227709

[63] Pascoe MC Thompson DR Ski CF. Yoga, mindfulness-based stress reduction and stress-related physiological measures: A meta-analysis[J]. Psychoneuroendocrinology. (2017) 86:152–68. doi: 10.1016/j.psyneuen.2017.08.008 28963884

[64] Streeter CC Whitfield TH Owen L Rein T Karri SK Yakhkind A . Effects of yoga versus walking on mood, anxiety, and brain GABA levels: a randomized controlled MRS study[J]. J Altern Complement Med. (2010) 16:1145–52. doi: 10.1089/acm.2010.0007 20722471 PMC3111147

[65] Field T Diego M Delgado J Medina L. Yoga and social support reduce prenatal depression, anxiety and cortisol[J]. J Bodyw Mov Ther. (2013) 17:397–403. doi: 10.1016/j.jbmt.2013.03.010 24138994

[66] Fox KCR Nijeboer S Dixon ML Fox KC Floman JL Ellamil M . Is meditation associated with altered brain structure? A systematic review and meta-analysis of morphometric neuroimaging in meditation practitioners[J]. Neurosci Biobehav Rev. (2014) 43:48–73. doi: 10.1016/j.neubiorev.2014.03.016 24705269

[67] Li W Garland EL McGovern P O’Brien JE Tronnier C Howard MO. Mindfulness-oriented recovery enhancement for internet gaming disorder in US adults: A stage I randomized controlled trial[J]. Psychol Addict Behav. (2017) 31:393–402. doi: 10.1037/adb0000269 28437120 PMC5468481

[68] Chen P Wang D Shen H Yu L Gao Q Mao L . Physical activity and health in Chinese children and adolescents: expert consensus statement (2020). Br J Sports Med. (2020) 54:1321–31. doi: 10.1136/bjsports-2020-102261 32471813 PMC7606574

[69] Du Z Zhang X. Analysis of the mediating effects of self-efficacy and self-control between physical activity and internet addiction among Chinese college students[J]. Front Psychol. (2022) 13:1002830. doi: 10.3389/fpsyg.2022.1002830 36211939 PMC9539857

[70] Hu J. Neuropsychological Mechanisms and Evidence of Mindfulness-Based Interventions for Addiction. J Educ Human Soc Sci. (2023) 8:904–908. doi: 10.54097/ehss.v8i.4379

[71] Matei D Trofin D Iordan DA Onu I Condurache I Ionite C . The Endocannabinoid System and Physical Exercise. Int J Mol Sci. (2023) 24:1989. doi: 10.3390/ijms24031989 36768332 PMC9916354

[72] Sleiman SF Henry J Al-Haddad R . Exercise promotes the expression of brain derived neurotrophic factor (BDNF) through the action of the ketone body β-hydroxybutyrate. eLife. (2016) 5:e15092. doi: 10.7554/elife.15092 27253067 PMC4915811

[73] Khoury B Knäuper B Schlosser M Carrière K Chiesa A. Effectiveness of traditional meditation retreats: A systematic review and meta-analysis[J]. J Psychosom Res. (2016) 92:16–25. doi: 10.1016/j.jpsychores.2016.11.006 27998508

[74] Wong HY Mo HY Potenza MN Chan MNM Lau WM Chui TK . Relationships between severity of internet gaming disorder, severity of problematic social media use, sleep quality and psychological distress[J]. Int J Environ Res Public Health. (2020) 17:1879. doi: 10.3390/ijerph17061879 32183188 PMC7143464

[75] Tang YY Tang R Posner MI. Mindfulness meditation improves emotion regulation and reduces drug abuse[J]. Drug Alcohol Depend. (2016) 163:S13–8. 27306725 10.1016/j.drugalcdep.2015.11.041

[76] Zhang JY Ji XZ Meng LN Cai YJ. Effects of modified mindfulness-based stress reduction (MBSR) on the psychological health of adolescents with subthreshold depression: a randomized controlled trial[J]. Neuropsychiatr Dis Treat. (2019):2695–704. doi: 10.2147/NDT.S216401 31571885 PMC6758632

[77] Stevens MWR King DL Dorstyn D Dorstyn N Delfabbro PH. Cognitive–behavioral therapy for Internet gaming disorder: A systematic review and meta-analysis[J]. Clin Psychol Psychother. (2019) 26:191–203. doi: 10.1002/cpp.2341 30341981

[78] Garland EL Howard MO. Mindfulness-based treatment of addiction: current state of the field and envisioning the next wave of research[J]. Addict Sci Clin Pract. (2018) 13:1–14. 29669599 10.1186/s13722-018-0115-3 PMC5907295

[79] Yao YW Chen PR Chiang-shan RL . Combined reality therapy and mindfulness meditation decrease intertemporal decisional impulsivity in young adults with Internet gaming disorder[J]. Comput Human Behav. (2017) 68:210–6. doi: 10.1016/j.chb.2016.11.038

[80] Liu J Nie J Wang Y. Effects of Group Counseling Programs, Cognitive Behavioral Therapy, and Sports Intervention on Internet Addiction in East Asia: A Systematic Review and Meta-Analysis. Int J Environ Res Public Health. (2017) 14:1470–19. doi: 10.3390/ijerph14121470 29182549 PMC5750889

[81] Li S Qianjin W Tang C . Exercise-Based Interventions for Internet Addiction: Neurobiological and Neuropsychological Evidence. Front Psychol. (2020) 11:1296. doi: 10.3389/fpsyg.2020.01296 32670157 PMC7330165

[82] Chen H Dong G Li K. Overview on brain function enhancement of Internet addicts through exercise intervention: Based on reward-execution-decision cycle. Front Psych. (2023) 14:1094583. doi: 10.3389/fpsyt.2023.1094583 PMC9933907 36816421

[83] Brand M Young K Laier C Wölfling K Potenza MN. Integrating psychological and neurobiological considerations regarding the development and maintenance of specific Internet-use disorders: An Interaction of Person-Affect-Cognition-Execution (I-PACE) model. Neurosci Biobehav Rev. (2016) 71:252–266. doi: 10.1016/j.neubiorev.2016.08.033 27590829

[84] Zhu Y Chen H Li J Mei X Wang W. Effects of different interventions on internet addiction: a systematic review and network meta-analysis. BMC Psychiatry. (2023) 23:921. doi: 10.1186/s12888-023-05400-9 38066462 PMC10704804

