# Supplementary material for: The intervention effect of physical and mental exercise on young adults internet addiction disorder: a systematic review and Bayesian model analysis
Source: Front Public Health. 2025 Oct 2;13:1670168. doi: 10.3389/fpubh.2025.1670168 (PMC12528216; doi:10.3389/fpubh.2025.1670168)
Supplement: Supplementary file 1 [file Data_Sheet_1.docx]

This study employed a Bayesian random effects framework to evaluate dose-response associations between mind-body exercise interventions and IAD. The analysis is based on the "brms" package (version 4.3.1) in R, which provides a Bayesian hierarchical model that can nest effect sizes in the study to manage the dependency relationships between multiple effect sizes of the same group of participants.The intercept parameter adopts weak information prior (prior distribution of overall effect size μ [0,1], studying heterogeneity between Tau [0,1]). According to Harrell's modeling strategy, the normal likelihood method with the same linking function is used to model the SMD changes in IAD and linear and nonlinear terms (i.e. natural spline, 3 or 4 segments) are used to adjust weekly physical activity. The selection of an ideal fitting model was based on various fitting metrics (namely point estimates of expected logarithmic point prediction density alongside standard error [SE] values, quantity of effective parameters, and information standards from cross validation). For analyzing dose-response relationships, Monte Carlo simulations via Markov chains (MCMC) were employed, with reliability enhanced through the establishment of four separate chains. Given the model's intricate nature, between 2000-4000 computational cycles were allocated per chain to facilitate proper model stabilization. To enhance analytical accuracy, the initial 1000 computational cycles from each chain were excluded as a burn-in phase, thereby minimizing potential initialization bias. In addition, we adopted a refinement strategy of saving results every 40 iterations to optimize the data monitoring process and reduce storage requirements. Finally, the convergence and effectiveness of the model were evaluated by examining the potential scaling factor (PSRF<1.05) of the estimated parameters and visualizing the MCMC chain.
